# The Circ_35953 induced by the NF‐κB mediated the septic AKI via targeting miR‐7219‐5p/HOOK3 and IGFBP7 axis

**DOI:** 10.1111/jcmm.17731

**Published:** 2023-03-28

**Authors:** Yuqing Feng, Bohao Liu, Jinwen Chen, Huiling Li, Dongshan Zhang

**Affiliations:** ^1^ Department of Emergency Second Xiangya Hospital, Central South University Changsha Hunan China; ^2^ Emergency Medicine and Difficult Diseases Institute Second Xiangya Hospital, Central South University Changsha Hunan China; ^3^ Department of Ophthalmology, Second Xiangya Hospital Central South University Changsha Hunan China; ^4^ Department of Nephrology Second Xiangya Hospital, Central South University Changsha Hunan China; ^5^ Hunan Aerospace Hospital Changsha Hunan China

**Keywords:** apoptosis, CircRNA, septic AKI

## Abstract

A few studies suggested that CircRNAs were involved in the development of septic AKI. However，the role and regulation mechanism of CircRNA_35953 in septic AKI remains unclear. Here, we found that Circ_35953 was induced by LPS via activation of NF‐κB signal in BUMPT cells. Functionally, Circ_35953 mediated the LPS induced the apoptosis in BUMPT cells. Moreover, we demonstrated that Circ_35953 sponged miR‐7219‐5p to upregulate the expression of HOOK3 and IGFBP7. Finally, we verified that knock down of Circ_35953 alleviated the progression of CLP‐induced AKI via targeting the miR‐7219‐5p/HOOK3 and IGFBP7 signal. Collectively, the data suggested that Circ_35953 /miR‐7219‐5p/HOOK3 and IGFBP7 axis mediated the septic AKI, which also revealed a potential mechanism of septic AKI.

## 1 INTRODUCTION

Sepsis, a life‐threatening clinical syndrome, is the key cause of acute kidney injury (AKI) and responsible for nearly half of all AKI patients.^1^, ^2^, ^3^ Recent more studies focused on the mechanism of SA‐AKI (septic AKI),^4^ the pathophysiological mechanism of it remains unknown, which leads to the no available and nonspecific therapy. Hence, it is the key step to unravel the pathophysiological mechanism of SA‐AKI progression for the development of effective therapeutic way.^2^

Circular RNAs (circRNAs), a class of non‐coding RNAs that do not have 5′ end caps or 3′ end poly (A) tails, has cell and tissue‐specific expression patterns.^5^ Mechanistically, circRNAs usually performed the pivotal biological function via multiple ways of microRNA sponges, translation templates, protein regulators and gene expression regulators.^6^, ^7^, ^8^ Furthermore, circRNAs have been implicated in diseases such as diabetes mellitus, neurodegenerative, ocular diseases, cardiovascular and cancer.^9^, ^10^, ^11^, ^12^, ^13^ More recent studies reported that CircRNAs was involved in the progression of SA‐AKI. For example, several CircRNAs of CIRC‐Ttc3, CircTLK1, Circ_0114428, CircHIPK3, circ‐FANCA and circ‐BNIP3L mediated the progression of SA‐AKI.^14^, ^15^, ^16^, ^17^, ^18^, ^19^ By the contrast, CircRNAs of CircVMA21, Circ_0068888 and Circ_0091702 protested against the development of SA‐AKI.^20^, ^21^, ^22^, ^23^ The above‐mentioned findings revealed the function of parts of cirRNAs. The Circ_35953 was one of circRNAs, localized in the position of cttnbp2nl. The role and mechanism of it in SA‐AKI remains largely unknown.

In current study, we demonstrated that both LPS and CLP induced the expression of Circ_35953 in vitro and vivo. The Circ_35953 mediated the LPS‐induced apoptosis in the BUMPT cells. Mechanistically, Circ_35953 sponged endogenous miR‐7219‐5p to increase the expression of HOOK3 and IGFBP7. Finally, silencing of Circ_35953 attenuated the progression of CLP‐induced AKI. Our findings found a novel regulation mechanism of Circ_35953 in SA‐AKI progression.

## 2 MATERIALS AND METHODS

### 2.1 Antibodies and reagents

β‐actin (cat. no. ab8227), Caspase3 (cat. no. ab32351) and cleaved caspase3 (cat. no. ab32042) were purchased from Abcam. IGFBP7 (cat. no.19961‐1‐AP) and HOOK3 (cat. no.15457‐1‐AP) were purchased from Proteintech. Luciferase assay kit was obtained from BioVision (Milpitas). The fluorescein isothiocyanate (FITC) Annexin V Apoptosis Detection Kit I (cat. no. 556547) was obtained from BD Pharmingen (Franklin).

### 2.2 Cell culture and treatments

BUMPT cells were cultured in DMEM (Sigma‐Aldrich), supplemented with 10% foetal bovine serum (FBS) and 1% penicillin–streptomycin (10,000 U/mL and 10,000 g/mL, respectively), and incubated at 37°C in a humidified atmosphere of 5% CO_2_. BUMPT cells were treated with saline or LPS (50 mg/mL) for 0, 6, 12 and 24 h. MiR‐7219‐5p antagomir (100 nM), miR‐7219‐5p mimic (100 nM), Circ_35953 siRNA (100 nM), HOOK3 siRNA (100 nM), or negative control (Ruibo) were transfected into BUMPT cells using Lipofectamine 2000. (Life Technologies).

### 2.3 Luciferase reporter assays

For the assessment of the microRNA (miRNA) activity, miRNA target insertion of sites was inserted at the end of the firefly luciferase gene (luc2) of the pmirGLO dual‐luciferase miRNA target expression vector (Promega). The luciferase vectors of Circ_35953 (WT‐Luc‐ Circ_35953)，HOOK3‐3′ UTR (WT‐Luc‐HOOK3)，mutated Circ_35953 (MUT‐Luc‐Circ_35953)，and HOOK3 (MUT‐Luc‐ HOOK3) was established by Sangon Biotech. Sequence alignment analysis revealed that CircRNA_35953 WT contained the complementary strand to miR‐7219‐5p, we build CircRNA_35953 mutation so that they are not complementary pairs. Circ_35953‐WT‐5′‐cagCAAGCCTGAACTCCTGACACt‐3′, Circ_35953‐MT‐5′‐cagCAAGAACACACTCTCTGATAt‐3′, miR‐7219‐5p‐3′‐agaGUUGGGACUCGA‐GAUUGUGu‐5′. PGMLR‐TK luciferase vector expressing Renilla luciferase (RLuc) was used as an internal control. The pGMLR‐TK plasmid was co‐transfected with WT‐Luc‐Circ_35953 or MUT‐Luc‐Circ_35953 plus with or without miR‐7219‐5p mimics into BUMPT cells. After 48 h of transfection, the luciferase reporter assay was performed as previously described.^4^ A SpectraMaxM5 (Molecular Devices) was used to detect the gene reporter activity and normalized by the RLuc signal.

### 2.4 Animal Models

Male C57BL/6 mice (10–12 weeks of age) were obtained from Sippr‐BK Laboratory Animal Corporation, and bred with Sterile water and food in a specific pathogen‐free facility under a 12‐h light/12‐h dark cycle. All of animal experiments were approved by the Animal Ethical and Welfare Committee of the Second Xiangya Hospital, Central South University (China). C57BL/6J mice were preinjected into tail vein with 15 mg/kg Circ_35953 siRNA or Circ_35953 siRNA‐cy3 (Ruibo) and then treated with cecum ligation and puncture (CLP) for AKI in line with the previously described.^24^ Briefly, Cut the skin, muscle layer and peritoneum layer by layer (the incision length is about 1 cm) at about 0.3 cm below the midpoint of the abdominal white line, take out the end of the cecum and place it outside the abdominal wall; Use 4–0 silk suture to ligate at 15 mm from the end of the cecum. After ligation, use no. 21 puncture needle to puncture two holes vertically into the cecum. The sham operation was considered as a control group. After 18 h, blood samples and renal tissues were collected to assess renal function and morphology analyses, respectively.

### 2.5 Renal function, morphological studies and apoptosis

The levels of BUN and creatinine were evaluated according to instruction of renal function examination KIT (Nanjing Jiancheng Bioengineering Institute, Jiangsu, China), haematoxylin and eosin staining was performed to assess morphology, and assessed by the renal injury score.^25^ TUNEL staining was also used to examine renal cell apoptosis, and then quantified by counting positive staining cells according to the previous described.^26^ An Olympus microscope equipped with UV epi‐illumination was applied to analysis the stained samples. FCM procedures were carried out according to the manufacturer's protocol.

### 2.6 Relative and absolute qPCR

Trizol Reagent (Invitrogen) was used to extract total RNA from BUMPT cells and kidney of C57BL/6J mice according to the manufacturer's procedure. Total RNA (40 ng) was reverse transcribed using Moloney murine leukaemia virus (M‐MLV) reverse transcriptase (Invitrogen). Real‐time qPCR was performed to examine the expression levels of miRNA, mRNA and circRNA using Bio‐Rad iQ SYBR Green Supermix with Opticon (MJ Research), according to the manufacturer's protocol. ∆Ct values were applied to carry out the relative quantification. The absolute quantification was performed according to a standard curve.

### 2.7 ChIP analysis

Chromatin immunoprecipitation (ChIP) assay the binding site of NF‐κB interaction with promoter of Circ_35953 was performed using commercial kit (Millipore). RT‐qRCR was used to analyse the precipitated DNA. The following specific primers were used for NF‐κB binding site detection: binding site 1, forward, AGCTGCAAGCTGCCATGT and reverse, ACCCTAAACCTGCTTACTGTAGACCTT;binding site2, forward, TCTGAATCTTCCAGTGTGGCTCCT and reverse, ATCTCCAACTGGCCCTATTTGTCG.

### 2.8 Immunoblot analysis

Equal amounts of proteins were separated by SDS‐PAGE and then transferred to a nitrocellulose membrane (Amersham). The membrane was incubated with primary antibodies against HOOK3, IGFBP7, caspase3, cleaved caspase3 and β‐actin followed by incubation with secondary antibody. β‐actin was used as an internal loading control.

### 2.9 Fluorescence in situ hybridisation (FISH)

The fluorescence probes of Circ_35953 and miR‐7219‐5p were synthesized by Ruibo, U6 and 18S were considered as nucleus and cytoplasmic control, respectively. Briefly, the slides of BUMPT cells and mice kidney were hybridized overnight with respective probes and subsequently stained with 4′,6‐diamidino‐2‐phenylindole (DAPI). A laser‐scanning confocal microscope was applied for fluorescence imaging analysis.

### 2.10 Statistical analyses

Two‐tailed Student's *t* tests were used for comparing two groups. One‐way anova was performed for multiple group comparison. Quantitative data were expressed as mean ± standard deviation (SD). The Spearman rank correlation coefficient was used to assess the correlations between variables. All statistical analyses were carried out with the SPSS package (SPSS) and GraphPad Prism software (GraphPad Prism Software). *p* < 0.05 was considered statistically significant.

## 3 RESULTS

### 3.1 Circ_35953 is induced by LPS and CLP in vitro and vivo

**FIGURE 1:**
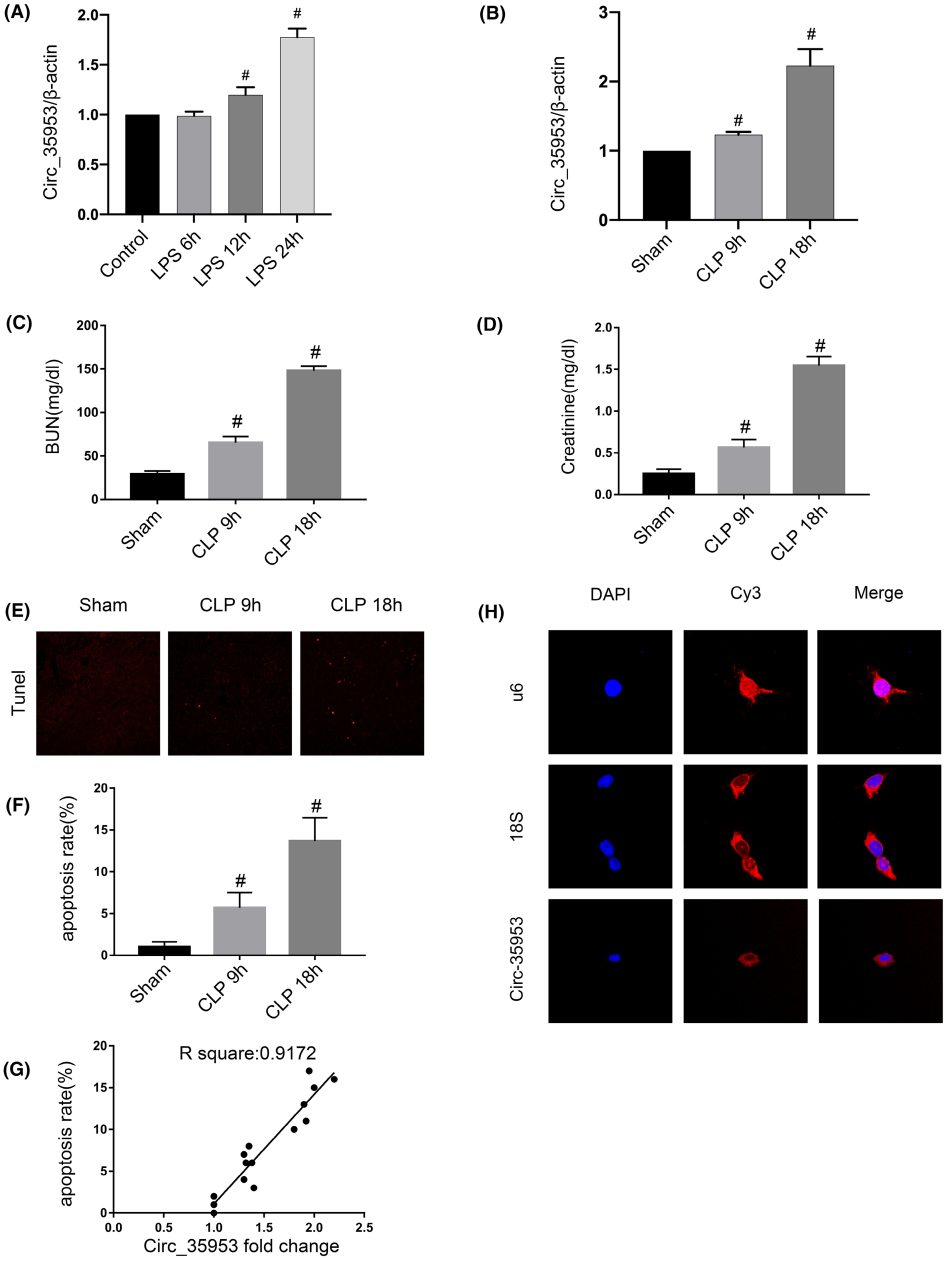
Circ_35953 was induced by LPS. BUMPT cells was treated with 300 μg/mL LPS for 6, 12 and 24 h. (A) RT‐qPCR analysis of the expression levels of Circ_35953 in cells. C56BL/6 mice were treated with CLP 9 h and 18 h. (B) RT‐qPCR analysis of the expression levels of Circ_35953 in kidney. (C, D) Time‐dependent increase of serum creatinine and BUN in CLP‐induced septic mice. (E) Representative images of TUNEL staining. (F) Analysis of TUNEL staining (G) Correlation analysis of Circ_35953 expression in retinal tissue with CLP‐induced apoptosis. (H) RNA‐FISH detection of intracellular localisation of Circ_35953 in BUMPT cells. Scale bar: 100 μm. #*p* < 0.05, LPS 12 h, LPS 24 h or CLP 9 h, CLP 18 h group versus Control or Sham group.

We explored whether Circ_35953 was induced by LPS and CLP in BUMPT cells and C57BL/6 mice, respectively. Here, the RT‐qPCR analysis results showed that the expression of Circ_35953 was induced by LPS at 12 h, and reached a peak at 24 h (Figure 1A). Furthermore, the RT‐qPCR analysis results also indicated that the expression of Circ_35953 was increased by CLP at 9 h, and attained a peak at 18 h (Figure 1B). The results of renal function and TUNEL staining (Figure 1E,F) showed that CLP‐induced the increasing of levels of BUN, Creatinine and apoptosis at 9 h, and reached a peak at 18 h (Figure 1C,D). The correlation analysis indicated that the renal cell apoptosis rate is highly associated with the expression of Circ_35953 foldchange (*R* = 0.9172; Figure 1G). The fluorescent in situ hybridisation (FISH) analysis indicated that Circ_35953 is in the cytoplasm of BUMPT cells (Figure 1H). The data showed that Circ_35953 was associated with the apoptosis.

### 3.2 NF‐κB mediated the expression of Circ_35953 during LPS treatment

**FIGURE 2:**
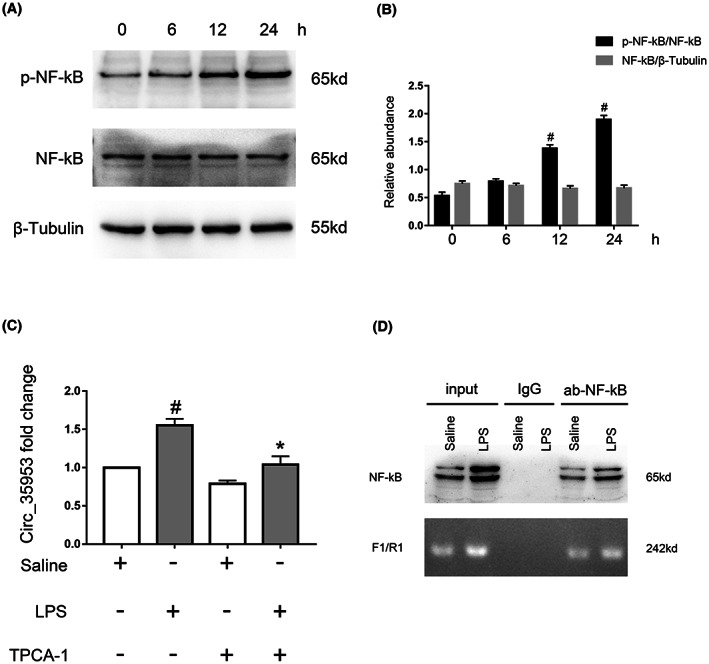
NF‐κB‐mediated the LPS‐induced the expression of Circ_35953. (A, B) Time‐dependent activation of NF‐κB signalling in LPS‐induced BUMPT cells. #*p* < 0.05, LPS 12 or 24 h group versus 0 h group (C) RT‐qPCR analysis of Circ_35953 expression in LPS‐induced BUMPT cells with pretreatment with NF‐κB inhibitor. (D) ChIP assays represent the binding sites of NF‐κB interaction with the gene promoter of Circ_35953. Data are expressed as mean ± SD (*n* = 6). #*p* < 0.05, LPS group versus saline group; *p < 0.05, TPCA‐1 with LPS group versus LPS group.

To investigate the potential regulation mechanism of the expression of Circ_35953 caused by the LPS treatment, we focused on the NF‐κB signal pathway. First, the immunoblot results showed that LPS induced the activation of NF‐κB in BUMPT cells at 12 h and reached a peak at 24 h (Figure 2A,B). Second, we found that two potential NF‐κB binding sites existed at the gene promoter of Circ_35953 by JASPAR Database (http://jaspar.genereg.net/), and then named as sites 1 and 2 (Table 1). Third, RT‐qPCR analysis demonstrated that TPCA‐1, a specific NF‐κB inhibitor, markedly suppressed the LPS‐induced the expression of Circ_35953 in BUMPT cells (Figure 2C). Finally, the ChIP assay indicated that LPS enhanced the binding of NF‐κB and the site 2 but not site 1 sequence of the gene promoter of Circ_35953 (Figure 2D). However, we found that knockdown of Circ‐35953 did not affect the activation of NF‐κB (Figure S3). The data suggested that NF‐κB directly upregulates the expression of Circ_35953 during LPS treatment.

**TABLE 1:** Predicted NF‐kB binding sites in mouse circ_35953 gene promoter.

^circ_35953^	^Score^	^Start^	^End^	^Sequence^
Site 1	7.271	1455	1464	GGGACTGCTC
Site 2	7.156	1774	1783	GGGAATCTCA

### 3.3 Knockdown of Circ_35953 expression ameliorated LPS induced BUMPT cell apoptosis

**FIGURE 3:**
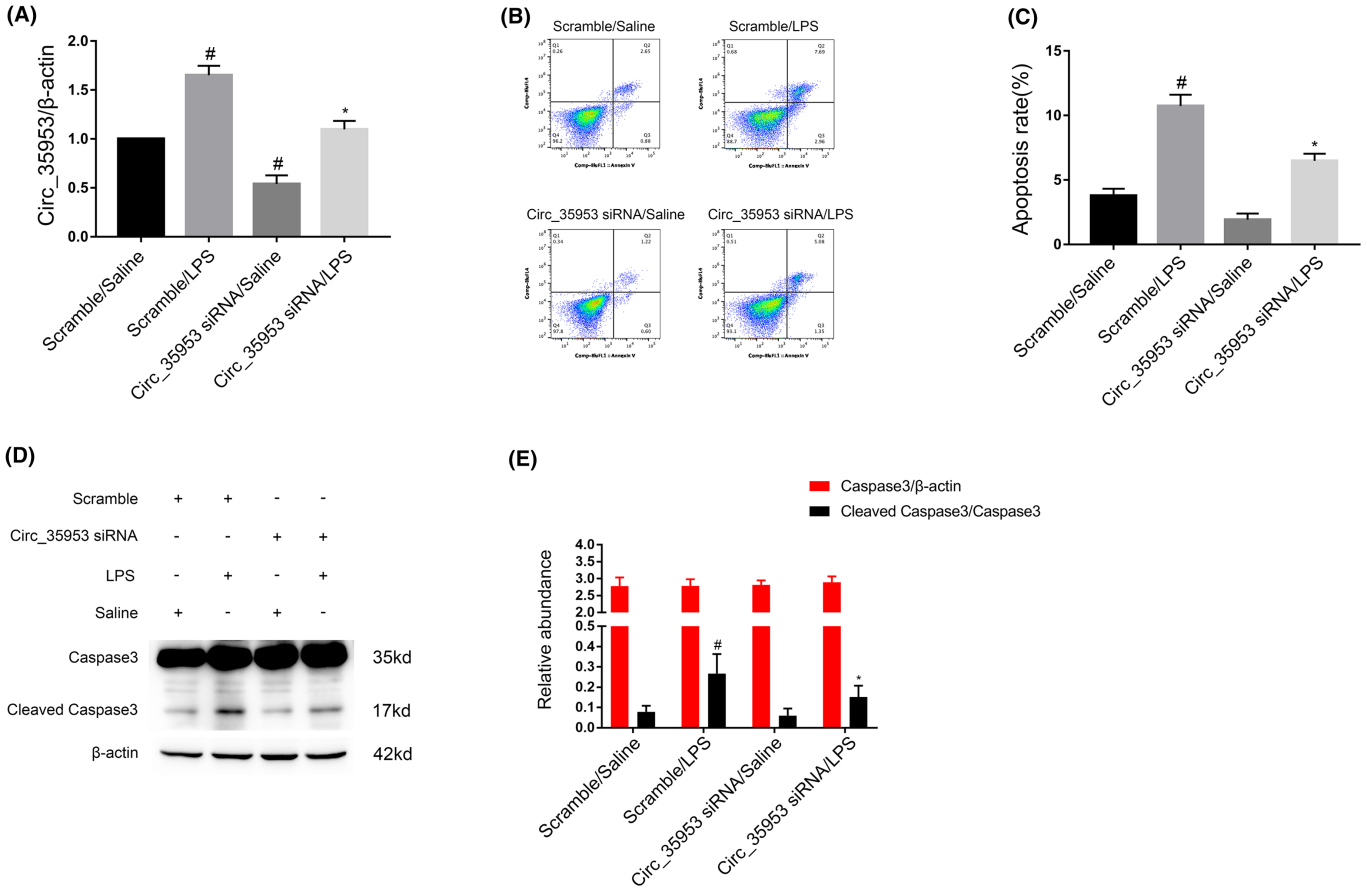
Silencing Circ_35953 inhibited LPS‐induced cells apoptosis. BUMPT cells were transfected with 100 nM Circ_35953 siRNA or scramble and then treated with or without 300 μg/mL LPS for 24 h. (A) RT‐qPCR analysis the expression levels of Circ_35953. (B, C) Flow cytometry analysis the apoptosis of BUMPT cells. (D) Western blot analysis of caspase3 and cleaved caspase3. (E) Densitometric analysis of immunoblot bands. Data are expressed as mean ± SD (*n* = 6). #*p* < 0.05, scramble with LPS versus scramble with saline group; *p < 0.05, Circ_35953 siRNA with LPS group versus scramble with LPS group.

Although the above‐mentioned data suggested that Circ_35953 might be involved apoptosis induced by LPS in BUMPT cells. The RT‐qPCR analysis showed that Circ_35953 siRNA significantly inhibited LPS‐induced Circ_35953 expression in BUMPT cells (Figure 3A). Additionally, the flow cytometry (FCM) results found that Circ_35953 siRNA noticeably suppressed the apoptosis caused by LPS in BUMPT cells (Figure 3B,C). The immunoblot detection results of cleaved caspase3 further the finding of FCM (Figures 3D,E). These data verified that Circ_35953 is an apoptosis inducer during LPS treatment.

### 3.4 Overexpression of Circ_35953 enhanced apoptosis caused by LPS in BUMPT cells

**FIGURE 4:**
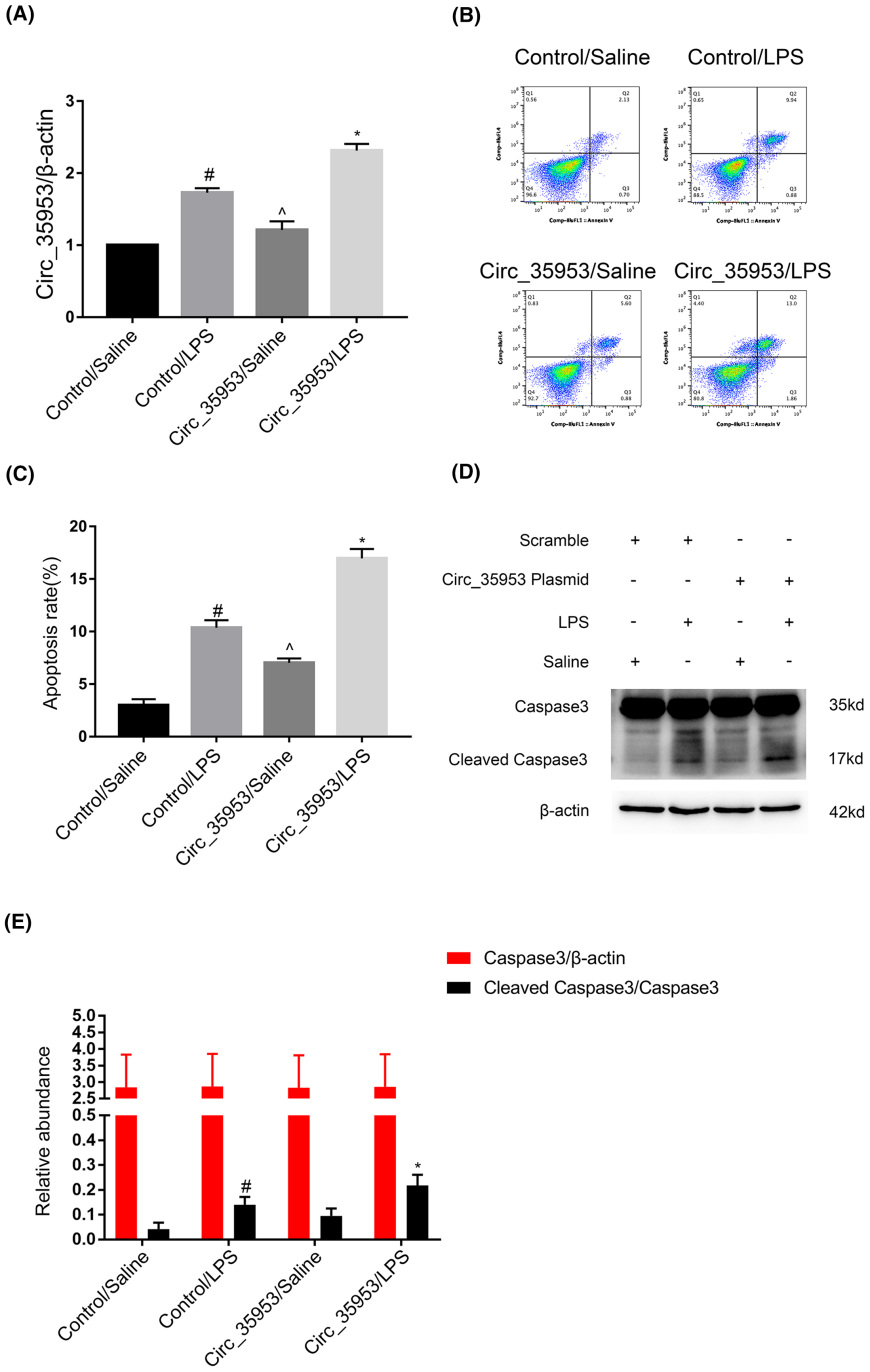
Circ_35953 accelerated LPS‐induced cells apoptosis. BUMPT cells were transfected with Circ_35953 plasmid or control and then with or without 300 μg/mL LPS for 24 h. (A) RT‐qPCR analysis the expression levels of CircRNA_35953. (B, C) FCM analysis BUMPT cells apoptosis. (D, E) Densitometric measurement of western blot bands for caspase3 and cleaved caspase3. Data are expressed as mean ± SD (*n* = 6). #*p* < 0.05, Control with LPS group versus Control with saline group; ^*p* < 0.05, Circ_35953 plasmid with Saline group versus Circ_35953 plasmid with LPS group; *p < 0.05, Circ_35953 plasmid with LPS group versus Control with LPS group.

To confirm the effect of Circ_35953 on apoptosis, the Circ_35953 expression vector was transfected into BUMPT cells, and then treated by LPS for 24 h. RT‐qPCR analysis showed that Circ_35953 overexpression enhanced the expression of it under basic and LPS treatment (Figure 4A). Moreover, the FCM results demonstrated that Circ_35953 overexpression enhanced the apoptosis caused by LPS in BUMPT cells (Figures 4B,C), which was further demonstrated by the immunoblot analysis results of the cleaved caspase3 (Figures 4D,E). The data supported the finding of Circ_35953 knockdown experiments, and further confirmed that Circ_35953 is an apoptosis driver during LPS treatment.

### 3.5 Circ_35953 sponged the miR‐7219‐5p

**FIGURE 5:**
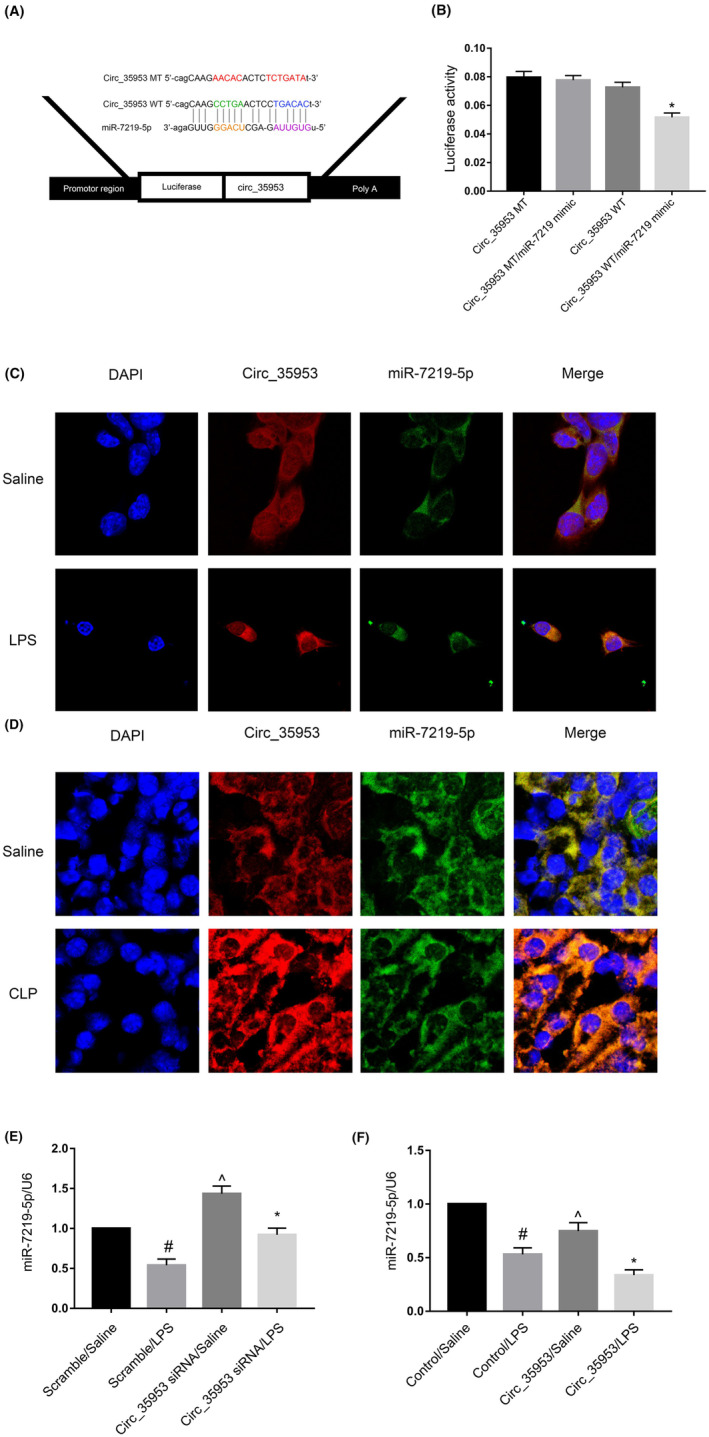
Circ_35953 directly binds to miR‐7219‐5p. (A) Sequence alignment analysis showed miR‐7219‐5p binding site in the Circ_35953 sequence. (B) Dual luciferase reporter detected relative luciferase activities in BUMPT cells after co‐transfection with Circ_35953‐WT or Circ_35953‐MUT and miR‐7219‐5p or scramble. *p < 0.05, Circ_35953 WT/miR‐7219 mimic group versus Circ_35953 WT and Circ_35953 MT/miR‐7219 mimic group. (C, D) RNA‐FISH detected intracellular co‐localisation of Circ_35953 and miR‐7219‐5p in BUMPT cells and C57BL/6J mice CLP kidney samples. (E, F) RT‐qPCR analysed miR‐7219‐5p expression levels after Circ_35953 knockdown or overexpression and then with or without LPS treatment. Data are expressed as mean ± SD (*n* = 6). #*p* < 0.05, scramble with LPS group versus scramble with saline group; *p < 0.05, Circ_35953 siRNA or Circ_35953 plasmid with Saline group versus scramble with scramble or control with saline group; *p < 0.05, Circ_35953 siRNA or Circ_35953 plasmid with LPS group versus scramble or control with LPS group.

As we known, circRNAs perform as competitive endogenous RNAs (ceRNAs) to sponge miRNAs. We predicated that Circ_35953 contained the complementary sequence of miR‐7219‐5p by the RegRNA 2.0 software (Figure 5A). The luciferase reporter gene assay showed that miR‐7219‐5p mimic suppressed the luciferase activity of Circ_35953‐wildtype (WT) but not Circ_35953‐mutant (MUT) (Figure 5B). The colocalisation experiments found that Circ_35953 interact with miR‐7219‐5p in the cytosolic compartment of BUMPT cells and renal cells of mice kidney under basic and LPS or CLP treatment, respectively (Figure 5C,D). Finally, Circ_35953 knockdown reversed the expression of miR‐7219‐5p under untreated and LPS treatment; however, the Circ_35953 overexpression suppressed the expression of miR‐7219‐5p under untreated and LPS treatment (Figure 5E,F). Collectively, the data show that miR‐7219‐5p is a direct target of Circ_35953.

### 3.6 MiR‐7219‐5p mimic alleviated LPS‐induced apoptosis in BUMPT cells

**FIGURE 6:**
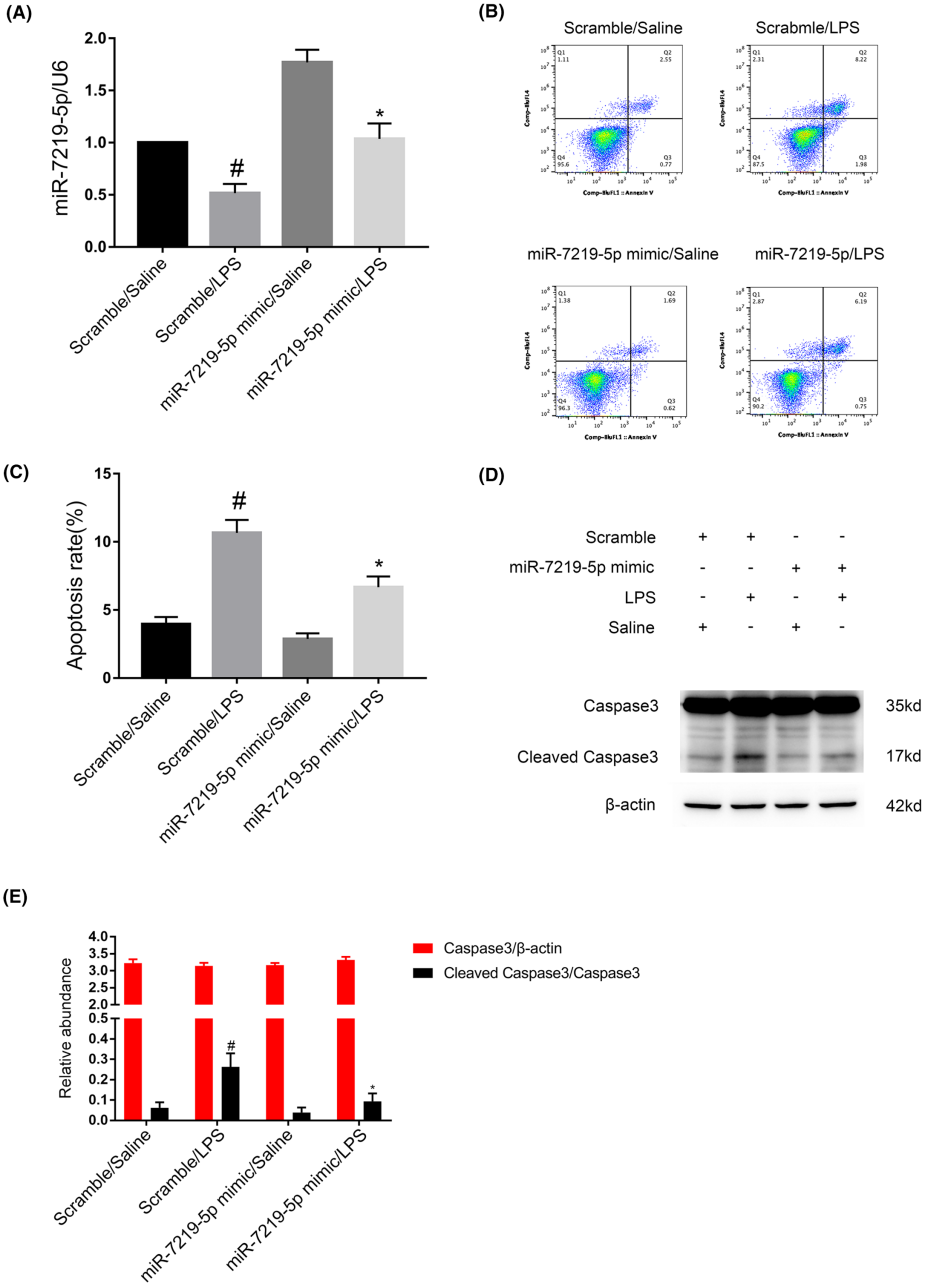
LPS‐induced BUMPT cell apoptosis was attenuated by miR‐7219‐5p mimics. BUMPT cells were transfected with 100 nM miR‐7219‐5p mimics or scramble and then treated with or without 50 mg/mL LPS for 24 h. (A) RT‐qPCR analysis of miR‐7219‐5p expression. (B, C) FCM analysis of BUMPT cell apoptosis. (D) Immunoblot analysis of cleaved caspase3 and caspase3. (E) Grey analysis of immunoblot bands. Data are expressed as mean ± SD (*n* = 6). #*p* < 0.05, scramble with LPS group versus scramble with saline group; *p < 0.05, miR‐7219‐5p mimics with LPS versus scramble with LPS group.

Here, the RT‐qPCR analysis showed that miR‐7219‐5p mimic markedly increased the expression of it under untreated and LPS treatment (Figure 6A). The FCM results showed that the miR‐7219‐5p mimic inhibited the LPS induced apoptosis in BUMPT cells (Figures 6B,C), which was verified by the immunoblot of the expression of cleaved caspase3 (Figures 6D,E) Hence, the data showed that miR‐7219‐5p protected from the apoptosis caused by LPS.

### 3.7 HOOK3, a target gene of miR‐7219‐5p, mediated LPS‐induced apoptosis

**FIGURE 7:**
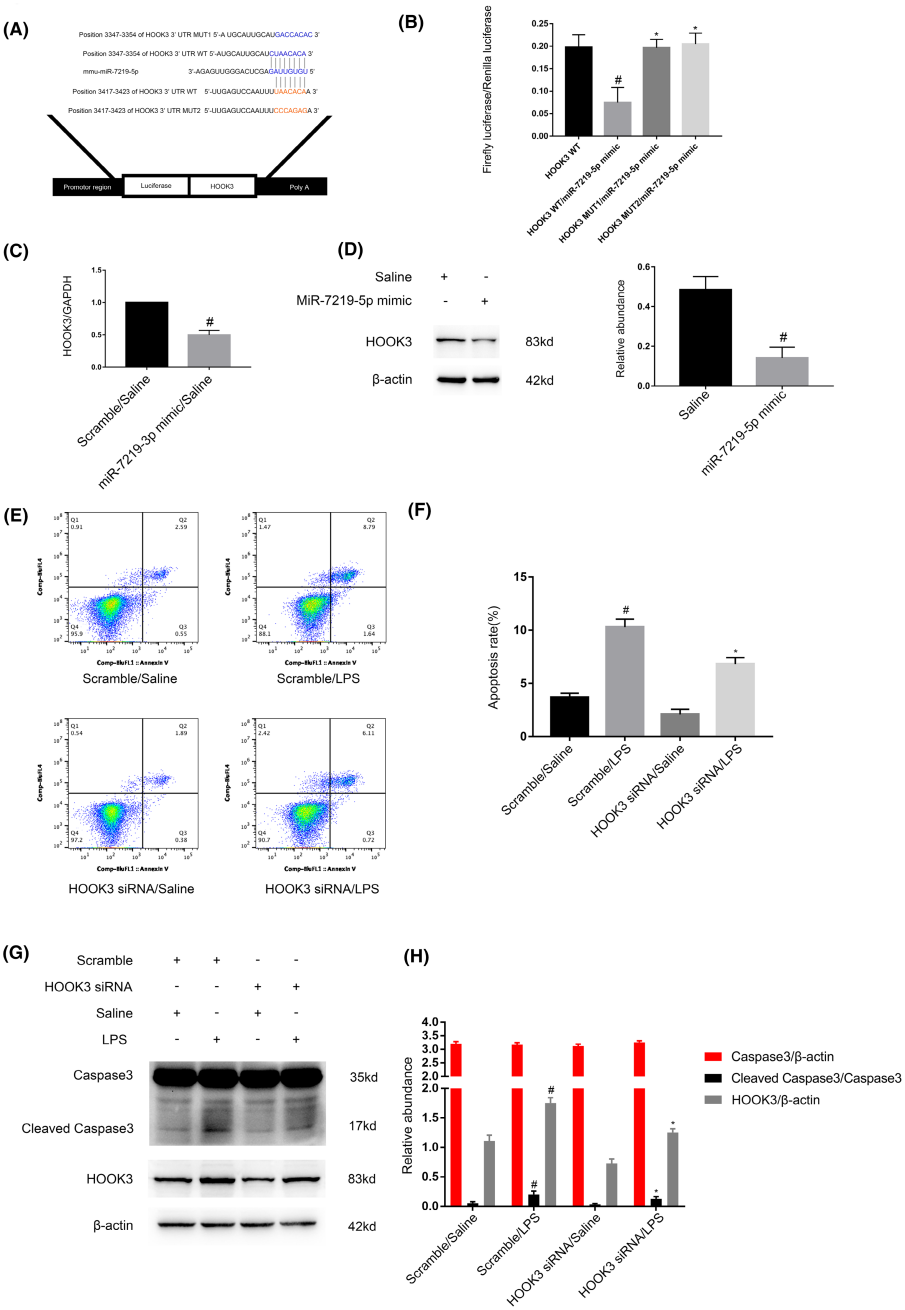
HOOK3 was identified as a target gene for miR‐7219‐5p. BUMPT cells were transfected with miR‐7219‐5p mimic or HOOK3 siRNA or scramble and then with or without 300 μg/mL LPS‐treated for 24 h. (A) TargetScan database predicted that miR‐7219‐5p complementary binding sites in the 3′UTR of HOOK3 mRNA. (B) Dual luciferase reporter detected relative luciferase activities in BUMPT cells after co‐transfection with HOOK3‐MUT1, HOOK3‐MUT2 or HOOK3‐WT and miR‐7219‐5p or scramble. #*p* < 0.05, HOOK3 WT/miR‐7219‐5p mimic group versus HOOK3/WT group; *p < 0.05 HOOK3 MUT1/miR‐7219‐5p mimic or HOOK3 MUT2/miR‐7219‐5p mimic group versus HOOK3 WT/miR‐7219‐5p mimic group. (C) RT‐qPCR analysis and immunoblot (D) revealed HOOK3 mRNA and protein expression levels. (E, F) Flow cytometry analysis of BUMPT cells apoptosis. #*p* < 0.05 miR‐7219‐5p mimic group versus Saline group. (G, H) Representative immunoblot images and densitometric analysis of HOOK3, caspase3 and cleaved caspase3. #*p* < 0.05, Scramble with LPS group versus Scramble with Saline group; *p < 0.05, HOOK3 siRNA with LPS group versus Scramble with LPS group. Data are expressed as mean ± SD (*n* = 6).

One study reported that Hook microtubule‐tethering protein 3(HOOK3) is an apoptosis inducer.^27^ We predicated that HOOK3 is a potential target gene of miR‐7219‐5p using the miRBase database (Figure 7A). The luciferase reporter gene assay demonstrated that the miR‐7219‐5p mimic inhibited the luciferase activity of HOOK3‐WT but not HOOK3‐MUT1 and HOOK3‐MUT2 (Figure 7B). The RT‐qPCR and immunoblot results indicated that the miR‐7219‐5p mimic markedly suppressed mRNA and protein levels of HOOK3 (Figure 7C,D). The FCM results showed that HOOK3 siRNA noticeably inhibited the LPS‐induced apoptosis in BUMPT cells (Figure 7E,F), which was confirmed by the immunoblot detection of the expression of HOOK3 and cleaved caspase‐3 (Figure 7G,H). Altogether, the data showed that HOOK3 was a direct target gene of miR‐7219‐5p.

### 3.8 MiR‐7219‐5p was a mediator of pro‐apoptosis effects of Circ_35953

**FIGURE 8:**
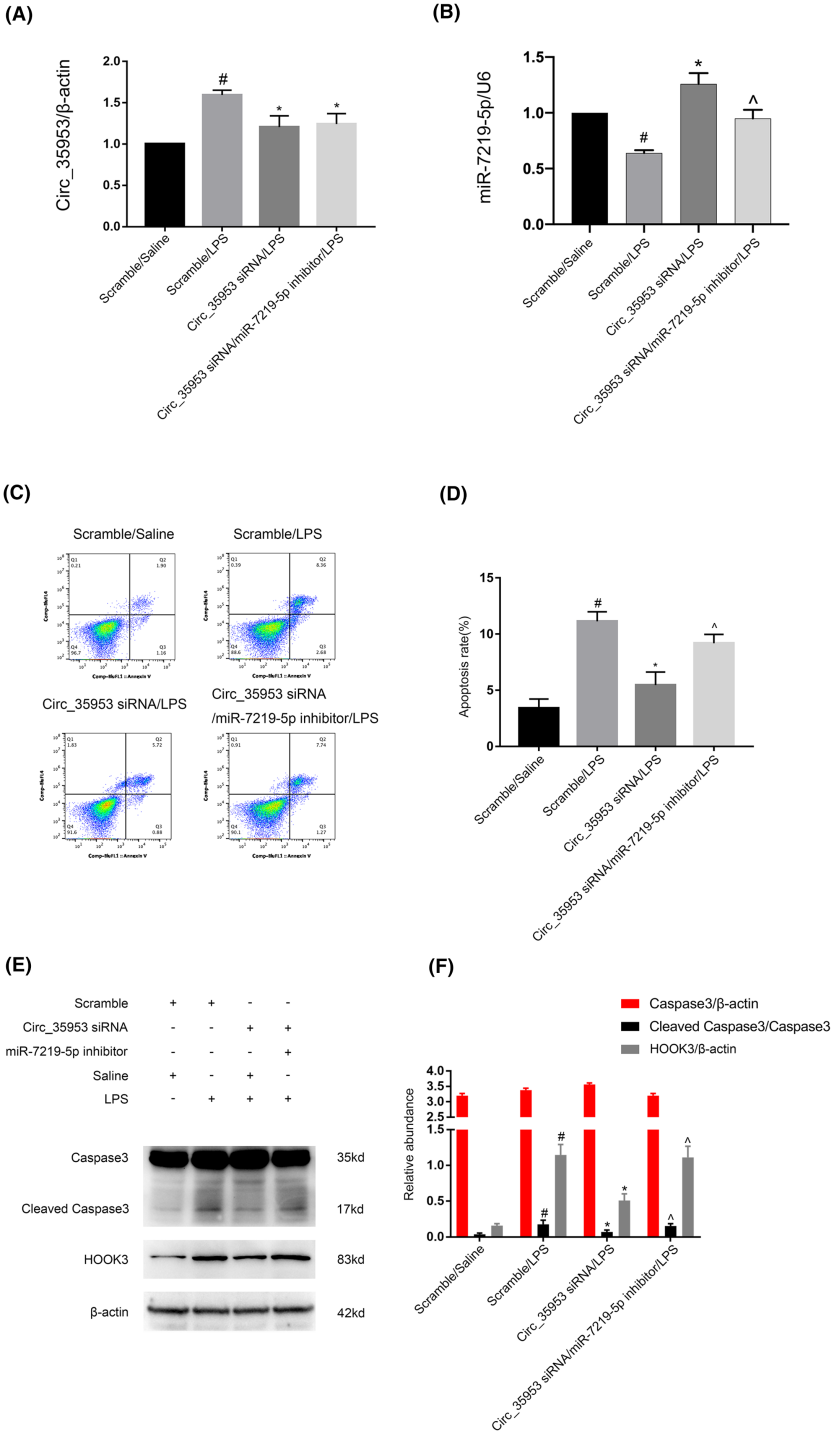
Inhibition of Circ_35953 attenuated the LPS‐induced BUMPT cell apoptosis, which was eeversed by the miR‐7219‐5p inhibitor. BUMPT cells were co‐transfected with Circ_35953 (100 nM) and anti‐miR‐7219‐5p or scramble and then treated with LPS for 24 h. (A, B) RT‐qPCR analysis of the expression levels of Circ_35953 and miR‐7219‐5p. (C, D) FCM analysis of BUMPT cell apoptosis. (E) Immunoblot analysis of cleaved caspase3 and caspase3. (F) Grey analysis of immunoblot bands. Data are expressed as mean ± SD (*n* = 6). #*p* < 0.05, scramble with LPS group versus scramble with Saline group; *p < 0.05, Circ_35953 siRNA with LPS group versus Scramble with LPS group; ^*p* < 0.05, Circ_35953 siRNA plus anti‐miR‐7219‐5p with LPS group versus Circ_35953 siRNA with LPS group.

To further confirm if miR‐7219‐5p mediates the proapoptotic effects of Circ_35953 during LPS treatment. RT‐qPCR analysis showed that Circ_35953 siRNA suppressed the expression of Circ_35953 while miR‐7219‐5p inhibitor also inhibited the expression of miR‐7219‐5p (Figure 8A,B). The FCM analysis showed that Circ_35953 siRNA alleviated the LPS‐induced apoptosis, which was reversed by the miR‐7219‐5p inhibitor (Figure 8C,D). Immunoblot analysis of cleaved caspase3 and HOOK3 further verified the findings of FCM (Figure 8E,F). The data confirmed that Circ_35953 mediated the LPS‐induced apoptosis by targeting of the miR‐7219‐5p.

### 3.9 Circ_35953 knockdown suppressed the progression of AKI caused by CLP by regulation of the miR‐7219‐5p/HOOK3 and IGFBP7 axis

**FIGURE 9:**
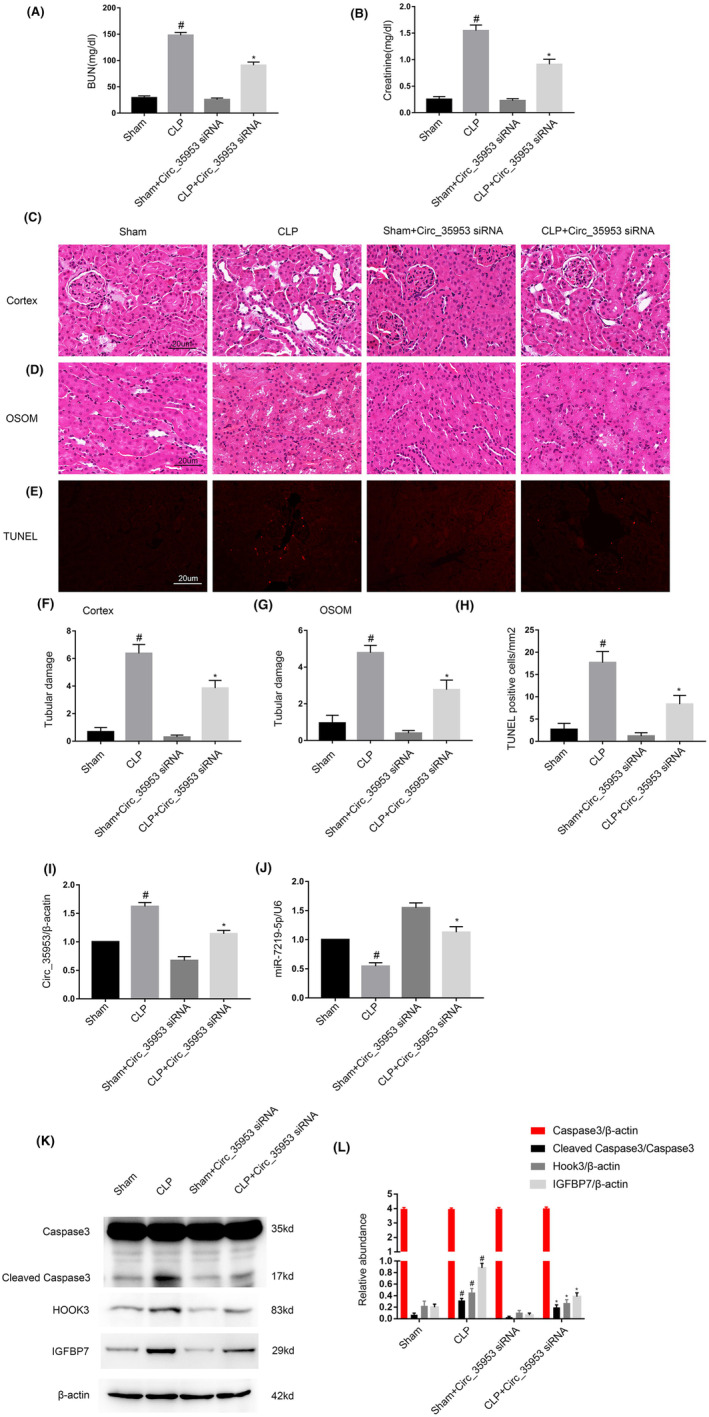
CLP‐induced AKI was alleviated by the reduced expression of Circ_35953 in Male C57BL/6 Mice. C57BL/6J mice were pretreated with the Circ_35953 siRNA via tail vein for 12 h and then subjected to CLP for 18 h or sham as control. (A, B) Blood serum was obtained for detection of nitrogen (BUN) (A) and creatinine (B) concentration. (C–E) The sections of kidney (cortex in C and OSOM in D) were stained with haematoxylin and eosin (H&E) and TUNEL (E). (F, G) Tubular damage scores of kidney cortex (F) and OSOM (G). (H) Counting of TUNEL‐positive cells. (I, J) RT‐qPCR analysis of the expression of Circ_35,953 and miR‐7219‐5p. (K) Immunoblot analysis of cleaved caspase3 and caspase3. (L) Grey analysis of immunoblot bands. Data are expressed as mean ± SD (*n* = 6). Scale bar: 100um. #*p* < 0.05, CLP group versus Sham group; *p < 0.05, Circ_35953 siRNA with CLP group versus CLP group. Original magnification, ×200.

To confirm the function of Circ_35953 in CLP‐induced AKI in vivo, C57BL/6J mice were preinjected with the Circ_35953 siRNA‐cy3 via tail vein for 12 h, and treated with CLP for 18 h. The fluorescence images showed circ_35953‐cy3 siRNA was successfully transfected into tubular cells of mice kidney (Figure S4). The renal function results suggested that Circ_35953 knock down reduced the CLP‐induced the increasing of both BUN and creatinine (Figure 9A,B). Haematoxylin and Eosin staining showed that Circ_35953 knock down suppressed CLP‐induced renal tubular injury in the cortex and OSOM (outer stripe of the outer medulla) of mice kidney (Figure 9C–G). TUNEL staining indicated that Circ_35953 knock down suppressed CLP‐induced renal cell apoptosis (Figure 9E,H). RT‐qPCR results found that Circ_35953 knock down suppressed CLP‐induced the expression of Circ_35953 and reversed the CLP‐induced the suppression of miR‐7219‐5p (Figure 9I,J). The Immunoblot analysis demonstrated that CLP‐induced the expression of cleaved caspase3, HOOK3, and IGFBP7 was inhibited by the Circ_35953 knock down (Figure 9K,L). The data showed that Circ_35953/miR‐7219‐5p/HOOK3 axis mediated the CLP‐induced the progression of AKI.

### 3.10 NF‐κB inhibitor suppressed the progression of BUMPT apoptosis and SA‐AKI caused by LPS and CLP by regulation of the Circ_35953/miR‐7219‐5p/HOOK3 Axis

To confirm the function of NF‐κB in CLP‐induced AKI in vivo, C57BL/6J mice were injected with the TPCA‐1(NF‐κB inhibitor) via tail vein for 24 h, and treated with CLP for 18 h. The renal function results suggested that inhibited NF‐κB reduced the CLP‐induced the increasing of both BUN and creatinine (Figure S1). RT‐qPCR results found that inhibited NF‐κB suppressed CLP‐induced the expression of Circ_35953 and reversed the CLP‐induced the suppression of miR‐7219‐5p (Figure S1). Haematoxylin and Eosin staining showed that inhibited NF‐κB suppressed CLP‐induced renal tubular injury in the cortex and OSOM (outer stripe of the outer medulla) of mice kidney (Figure S1). TUNEL staining indicated that inhibited NF‐κB suppressed CLP‐induced renal cell apoptosis (Figure S1). The Immunoblot analysis demonstrated that CLP‐induced the expression of p‐ NF‐κB, NF‐κB, cleaved caspase3 and HOOK3 was inhibited by the inhibition of NF‐κB (Figure S1). The above‐mentioned data suggested that NF‐κB might be involved apoptosis induced by LPS in BUMPT cells. The RT‐qPCR analysis showed that TPCA‐1 significantly inhibited Circ_35953 and increased miR‐7219‐5p expression in BUMPT cells (Figure S2A,B). The immunoblot detection results of p‐ NF‐κB, NF‐κB, HOOK3, cleaved caspase3 and caspase3 (Figure S2C,D). These data verified that NF‐κB is an apoptosis inducer during LPS treatment.

### 3.11 IGFBP7, another target gene of miR‐7219‐5p, mediated LPS‐induced apoptosis

We predicated that IGFBP7 is another potential target gene of miR‐7219‐5p using the miRBase database (Figure S5A). The luciferase reporter gene assay demonstrated that the miR‐7219‐5p mimic inhibited the luciferase activity of IGFBP7‐WT but not IGFBP7‐MUT1 and IGFBP7‐MUT2 (Figure S5B). The RT‐qPCR and immunoblot results indicated that the miR‐7219‐5p mimic markedly suppressed mRNA and protein levels of IGFBP7 (Figure S5C,D). Immunoblot analysis of IGFBP7 (Figure S5F).

## 4 DISCUSSION

Recent studies suggested that several CircRNAs play a pivotal role in SA‐AKI.^14^, ^15^, ^16^, ^17^, ^18^, ^19^, ^20^, ^21^, ^22^, ^23^ However, the function of more CircRNAs needed to be investigated. In current study, we for the first time reported that Circ_35953 mediated the LPS‐induced the apoptosis in BUMPT cells. Moreover, Circ_35953 acted as a ceRNA to induce the apoptosis via targeting miR‐7219‐5p/HOOK3 and IGFBP7 axis, which was demonstrated by the LPS and CLP injury models in vitro and vivo. Collectively, we verified that Circ_35953 was one of drivers of SA‐AKI.

The studies indicated that the programmed cell death of tubular epithelial cells played a key role in SA‐AKI.^28^, ^29^, ^30^ CircRNAs were involved in the renal cell apoptosis. For example, the CIRC‐Ttc3, circVMA21, and Circ_0091702 suppressed LPS induced the tubular cell apoptosis.^14^, ^20^, ^21^, ^23^ However, circTLK1, circ_0114428, circHIPK3, and circ‐FANCA promoted LPS‐induced tubular cell apoptosis.^15^, ^16^, ^17^, ^18^ In present study, we found that Circ_35953 mediated the LPS‐induced the apoptosis in BUMPT cells, which was demonstrated by the knock down and overexpression of Circ_35953 experiments (Figures 3 and 4). Furthermore, the evidence was supplied by the vivo finding that silencing of Circ_35953 also attenuated the CLP‐induced the tubular cell apoptosis (Figure 9). Altogether, the data verified that Circ_35953 was an apoptosis driver in SA‐AKI.

How about the regulation mechanism of Circ_35953 for the tubular cell apoptosis? We verified that Circ_35953 located in the cytoplasm of BUMPT cells (Figure 1). Previous study reported that circRNAs in the cytoplasm could act as microRNA to regulate the expression of target mRNAs.^31^ The multiple ways of bioinformatics analysis, Dual‐luciferase, RNA‐FISH and RT‐qPCR demonstrated that the miR‐7219‐5p was a direct target of Circ_35953 (Figure 5). Collectively, the data strongly suggested that miR‐7219‐5p was a direct target of Circ_35953.

The function and target gene of miR‐7219‐5p remains unclear. Here, we for the first time demonstrated that miR‐7219‐5p mimics markedly suppressed the LPS‐induced apoptosis and the increasing of cleaved caspase3 using the detection of FCM and immunoblot (Figure 6). We further verified that HOOK3 mRNA is a direct target of miR‐7219‐5p by the miRtarget prediction website and dual luciferase reporter assay (Figure 7A,B). The results RT‐qPCR and western blot confirmed that miR‐7219‐5p noticeably suppressed the mRNA and protein expression of HOOK3 (Figure 7C,D). Knockdown HOOK3 decreased the BUMPT cells apoptosis and caspase3 activation in vitro (Figure 7E–H), which is consistent with the previous finding in cardiomyocyte apoptosis caused by the I/R.^27^ We also revealed that silence Circ_35953 expression decreased LPS‐induced renal cell apoptosis, and these effects were reversed by miR‐7219‐5p inhibitor (Figure 8). Moreover, Circ_35953 siRNA suppressed CLP‐induced kidney injury via miR‐7210‐5p axis (Figure 9).

NF‐κB is a protein family consisting of 5 dimers, RelA (p65), RelB, c‐Rel, p50 (generated from p105), and p52 (generated from p100), which can form a variety of homodimers or heterodimers. Normally, NF‐κB dimers are inactivated through interacting with the inhibitor of κB (IκB).^32^ As one of the most important components of the pathogenesis, systematic inhibition of NF‐κB affects the severity of AKI. In a disease model induced by folic acid, inhibition of NF‐κB mitigates AKI‐injury by reduction of RelA and NFκB2 activation.^33^ In our study, we found NF‐κB inhibitor suppressed the progression of BUMPT apoptosis and SA‐AKI caused by LPS and CLP (Figures S1 and S2).

HOOK3, the human hook microtubule tethering proteins family comprises,^27^ a well‐established dynein‐activating adaptor.^34^ Previous studies have reported that HOOK3 can serve as a fusion partner in gastrointestinal stromal tumour (GIST) and papillary thyroid carcinoma. The chimeric HOOK3‐FGFR1 fusion protein contains the coiled‐coil domain from HOOK3, indicating its potential leukaemogenesis role in EMS.^35^

IGFBP7, insulin‐like growth factor binding protein 7, a member of the insulin‐like growth factor (IGF)‐binding protein (IGFBP) family. Kashani et al.^36^ reported that IGFBP7 and TIMP‐2 were identified as septic AKI biomarkers. Furthermore, IGFBP7 mediated the podocyte apoptosis caused by high glucose.^37^ Interestingly, recent study verified that IGFBP7 promoted LPS‐induced renal proximal tubular cell apoptosis in septic AKI.^38^ Here, we found that IGFBP7 was another proapoptotic target gene of miR‐7219‐5p (Figure S5). Finally, we demonstrated that knockdown of Circ_35953 also suppressed the expression of IGFBP7 in mice CLP‐induced septic AKI model (Figure 9).

In summary, we found that Circ_35953 accelerates CLP‐induced AKI via the miR‐7219‐5p/HOOK3 and IGFBP7 axis, which presenting a new mechanism of SA‐AKI development.

## AUTHOR CONTRIBUTIONS

**Yuqing Feng:** Data curation (equal); formal analysis (equal); investigation (equal); writing – original draft (equal). **Bohao Liu:** Formal analysis (equal); investigation (equal); software (equal); writing – original draft (equal). **Jinwen Chen:** Funding acquisition (equal); investigation (equal). **Huiling li:** Funding acquisition (equal); methodology (equal); project administration (equal); resources (equal); supervision (equal); writing – review and editing (equal). **Dongshan Zhang:** Conceptualization (equal); data curation (equal); project administration (equal); resources (equal); supervision (equal); validation (equal); writing – review and editing (equal).

## FUNDING INFORMATION

The study was supported in part by a grant from National Natural Science Foundation of China (81870475, 82171088, 81570646). Key Project of Hunan provincial science and technology innovation (2020SK1014, 2021SK2034). China Hunan Provincial Science and Technology Department (project no. 2021sk4004) and the Natural Science Foundation of Hunan Province (2021JJ30935, 2021JJ30228).

## CONFLICT OF INTEREST STATEMENT

The authors declare no competing interests.

## CONSENT FOR PUBLICATION

All authors agree to publish in Journal of Cellular and Molecular Medicine.

## Supporting information


Figures

## Data Availability

The datasets used and/or analysed during the current study are available from the corresponding author on reasonable request.
