# Supplementary material for: The Circ_35953 induced by the NF‐κB mediated the septic AKI via targeting miR‐7219‐5p/HOOK3 and IGFBP7 axis
Source: J Cell Mol Med. 2023 Mar 28;27(9):1261–76. doi: 10.1111/jcmm.17731 (PMC10148060; doi:10.1111/jcmm.17731)
Supplement: Supplementary file 1 — Figures [file JCMM-27-1261-s001.docx]

**The Circ_35953 induced by the NF-κB mediated the septic AKI via targeting miR-7219-5p/HOOK3 and IGFBP7 axis**

**Supplementary Figure1**


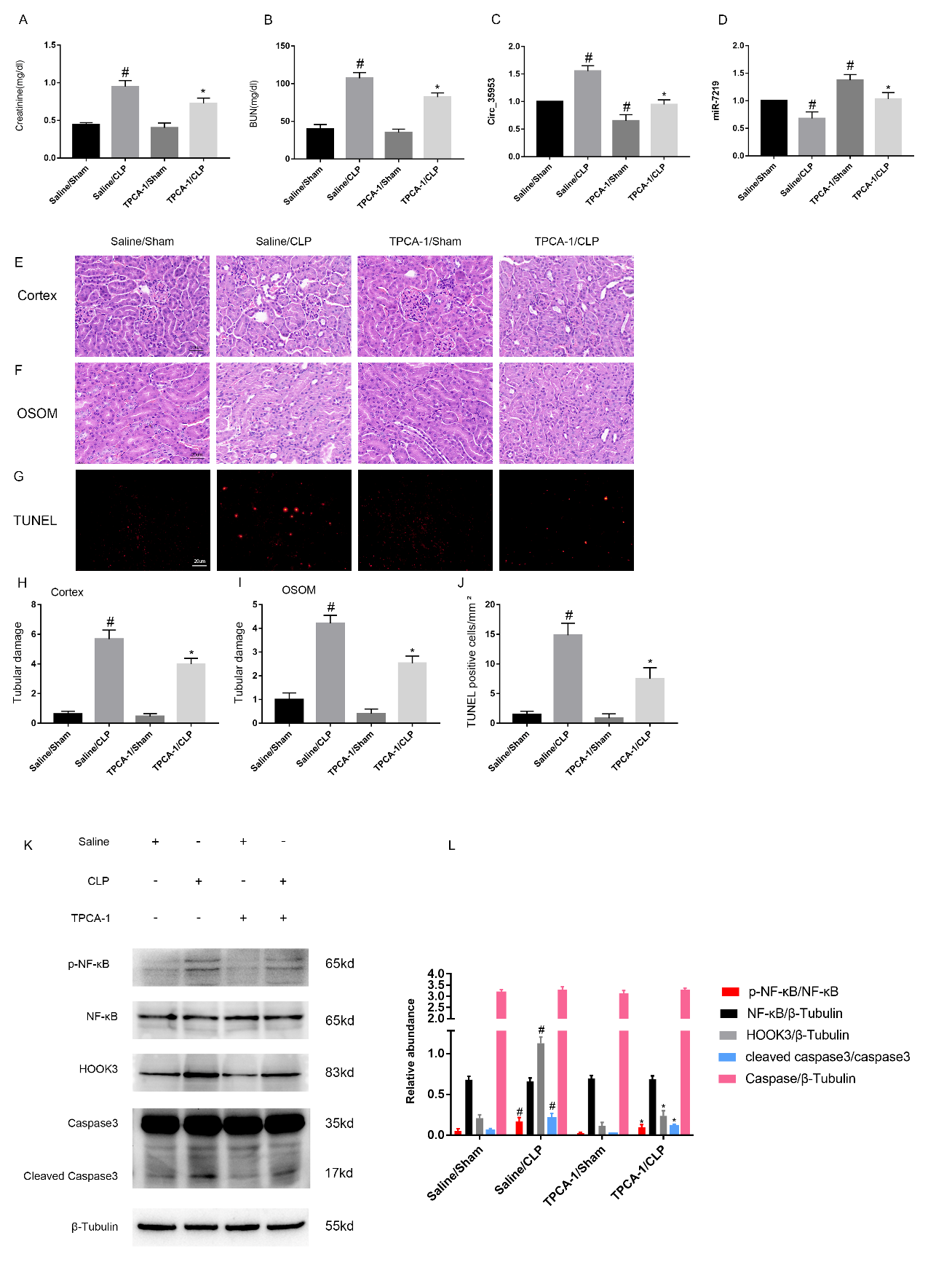


**Inhibition of NF-κB attenuates the progression of CLP induced AKI in mice via** **regulation of the Circ_35953/miR-7219-5p/HOOK3 Axis**

TPCA-1(NF-κB inhibitor) or Salinewas injected into male C57BL/6 mice through tail vein. After 12 hours, the mice were subjected to CLP for 18 h or sham as control. (A and B) Blood serum was obtained for detection of nitrogen (BUN) (A) and creatinine (B) concentration. (C and D) RT-qPCR analysis of the expression of Circ_35953 and miR-7219-5p. (E-G) The sections of kidney (cortex in E and OSOM in F) were stained with hematoxylin and eosin (H&E) and TUNEL (G). (H and I) Tubular damage scores of kidney cortex (H) and OSOM (I). (J) Counting of TUNEL-positive cells. (K) Immunoblot analysis of p- NF-κB, NF-κB, HOOK3, cleaved caspase3, and caspase3. (L) Gray analysis of immunoblot bands. Data are expressed as mean ± SD (n = 6). Scale bar: 20um. #*p < 0.05*, CLP group versus Sham group; **p < 0.05*, TPCA-1 with CLP group versus CLP group. Original magnification, x200.

**Supplementary Figure 2**


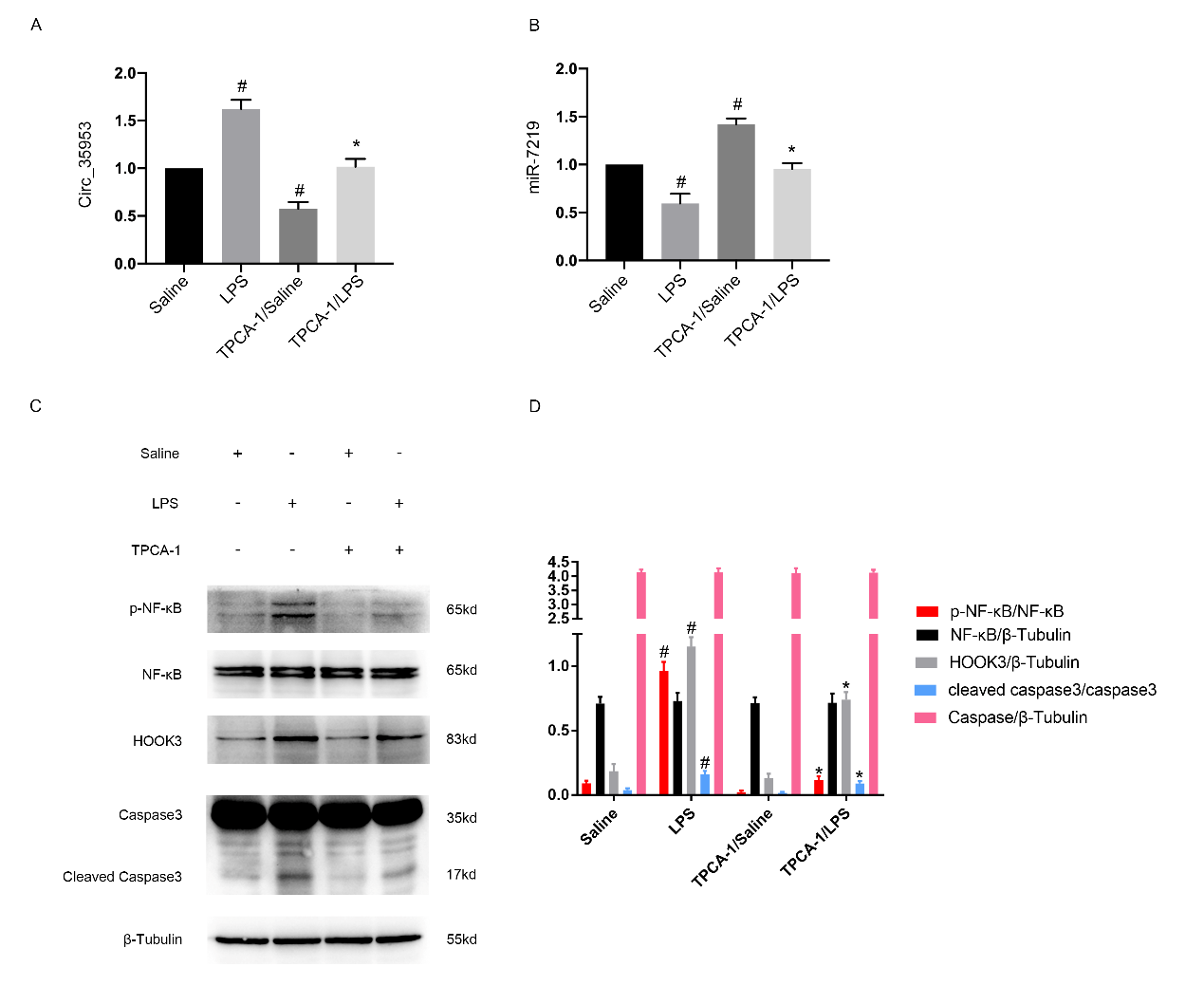


**TPCA-1 suppresses LPS-induced BUMPT cells apoptosis via** **regulation of the Circ_35953/miR-7219-5p/HOOK3 Axis**

BUMPT cells were treated with 100μM TPCA-1 and then treated with LPS. (A and B) RT-qPCR analysis of the expression of Circ_35953 and miR-7219-5p.(C) Immunoblot analysis of p-NF-κB, NF-κB, HOOK3, cleaved caspase3 and caspase3.(L) Gray analysis of immunoblot bands. Data are expressed as mean ± SD (n = 6). #p < 0.05, LPS group versus Sham group; *p < 0.05, TPCA-1 with LPS group versus LPS group.

**Supplementary Figure 3**


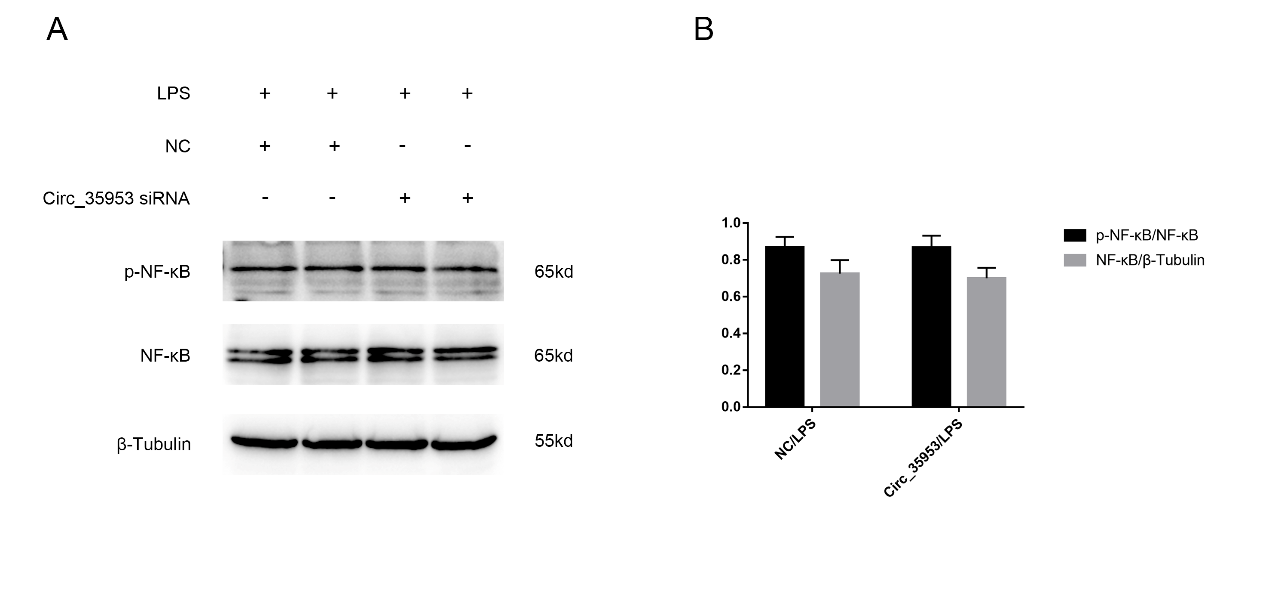


**Knockdown Circ-35953 does not affect the expression of p-NF-κB**

BUMPT cells were treated with 100nM Circ_35953 siRNA and then treated with LPS. (A) Immunoblot analysis of p-NF-κB, NF-κB. (B) Gray analysis of immunoblot bands. There is no significant change.

**Supplementary Figure 4**


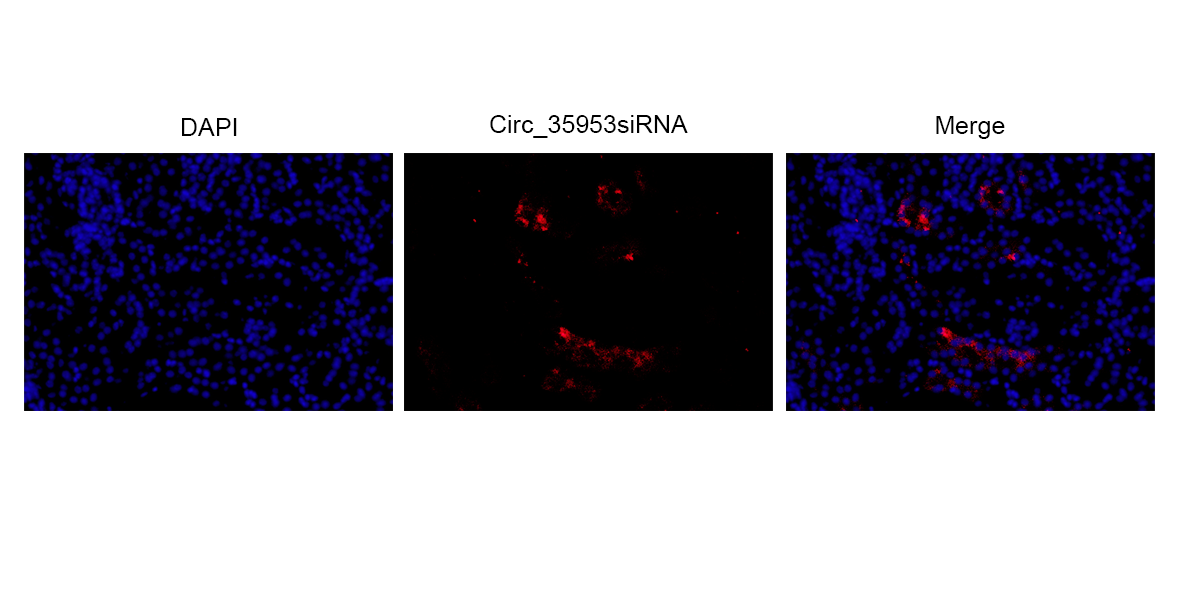


**Pre injection with circ_35953 siRNA-cy3 in mice**

Frozen section for Fluorescence images of transfection.

**Supplementary Figure 5**


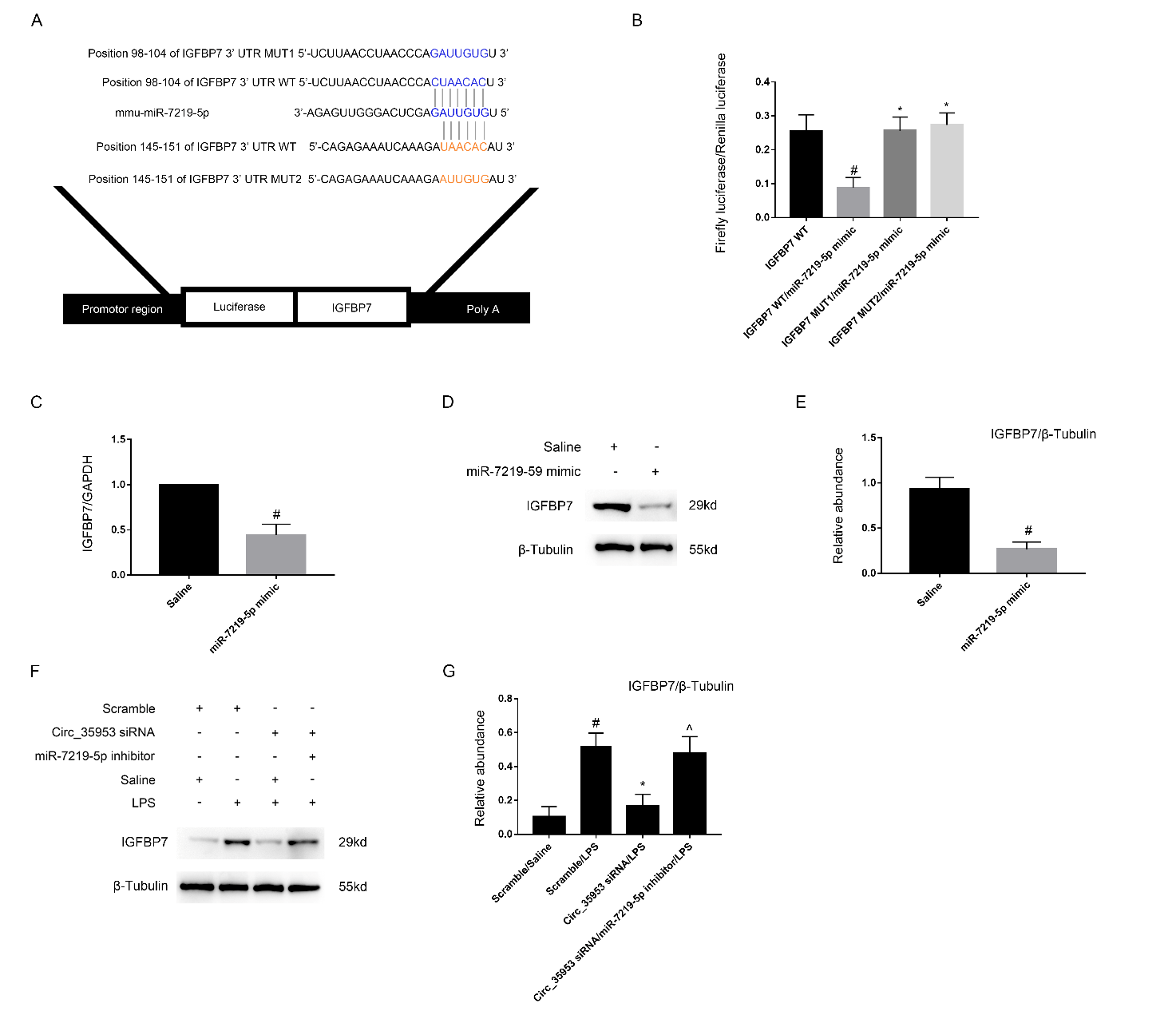


**IGFBP7, a target gene of miR-7219-5p, mediated LPS-induced apoptosis**

(A) TargetScan database predicted that miR-7219-5p complementary binding sites in the 3’UTR of IGFBP7 mRNA. (B) Dual luciferase reporter detected relative luciferase activities in BUMPT cells after co-transfection with IGFBP7-MUT1, IGFBP7-MUT2 or IGFBP7-WT and miR-7219-5p or scramble. #p < 0.05, IGFBP7 WT/miR-7219-5p mimic group versus IGFBP7/WT group; *p < 0.05 IGFBP7 MUT1/miR-7219-5p mimic and IGFBP7 MUT2/miR-7219-5p mimic group versus IGFBP7 WT/miR-7219-5p mimic group. (C) RT-qPCR analysis. (D and F) Immunoblot results of IGFBP7. (E and G) Gray analysis of immunoblot bands. Data are expressed as mean ± SD (n = 6). #p < 0.05, miR7219-5p mimic group versus Saline group or Scramble/LPS group versus Scramble/Saline group; *p < *0.05*, Circ_35953 siRNA/LPS versus Scramble/LPS; ^ *< 0.05*,Circ_35953 siRNA/miR-7219-5p inhibitor/LPS versus Circ_35953 siRNA/LPS.
